# Yu Ping Feng San, an Ancient Chinese Herbal Decoction, Regulates the Expression of Inducible Nitric Oxide Synthase and Cyclooxygenase-2 and the Activity of Intestinal Alkaline Phosphatase in Cultures

**DOI:** 10.1371/journal.pone.0100382

**Published:** 2014-06-26

**Authors:** Crystal Y. Q. Du, Roy C. Y. Choi, Tina T. X. Dong, David T. W. Lau, Karl W. K. Tsim

**Affiliations:** Division of Life Science and Centre for Chinese Medicine, The Hong Kong University of Science and Technology, Hong Kong SAR, China; Istituto Superiore di Sanità, Italy

## Abstract

Yu Ping Feng San (YPFS), a Chinese herbal decoction comprising Astragali Radix (AR; Huangqi), Atractylodis Macrocephalae Rhizoma (AMR; Baizhu), and Saposhnikoviae Radix (SR; Fangfeng), has been used clinically to treat inflammatory bowel diseases (IBD). Previously, we demonstrated a dual role of YPFS in regulating cytokine release in cultured macrophages. In this study, we elucidated the anti-inflammatory effect of YPFS that is mediated through modulating the expression of three key enzymes involved in IBD: inducible nitric oxide synthase (iNOS), cyclooxygenase-2 (COX-2), and intestinal alkaline phosphatase (IALP). In a lipopolysaccharide (LPS)-induced chronic-inflammation model of cultured murine macrophages, YPFS treatment suppressed the activation of iNOS and COX-2 expression in a dose-dependent manner. Conversely, application of YPFS in cultured small intestinal enterocytes markedly induced the expression of IALP in a time-dependent manner, which might strengthen the intestinal detoxification system. A duality of YPFS in modulating the expression of iNOS and COX-2 was determined here. The expression of iNOS and COX-2 in macrophages was induced by YPFS, and this activation was partially blocked by the NF-κB-specific inhibitor BAY 11-7082, indicating a role of NF-κB signaling. These YPFS-induced changes in gene regulation strongly suggest that the anti-inflammatory effects of YPFS are mediated through the regulation of inflammatory enzymes.

## Introduction

Yu Ping Feng San (YPFS) is composed of Astragali Radix (AR; Huangqi, the root of *Astragalus membranaceus* (Fisch.) Bunge or *Astragalus membranaceus* (Fisch.) Bunge var. *mongholicus* (Bunge) P.K. Hsiao), Atractylodis Macrocephalae Rhizoma (AMR; Baizhu, the rhizomes of *Atractylodes macrocephala* Koidz.), and Saposhnikoviae Radix (SR; Fangfeng, the roots of *Saposhnikovia divaricata* (Turcz.) Schischk.) in a weight ratio of 1∶2∶1. This herbal formula was first described in “*Dan Xi Xin Fa”* by Zhu Danxi in Yuan Dynasty (A.D. 1279–1368) of China. In accordance with traditional Chinese medicine (TCM) theory, YPFS is frequently being used to treat colds, flus and inflammation-associated diseases. Clinically, YPFS has been shown to produce beneficial immune-modulatory effects of preventing bacterial and viral infections. Recent studies revealed that YPFS exerts antiviral effects including effects against influenza virus, human respiratory syncytial virus, and severe acute respiratory syndrome (SARS) virus [1]–[5], as well as curative effects in inflammation-associated diseases including allergic rhinitis [6], [7] and asthma [8].

Our recent studies showed that YPFS is a potent immune stimulator that activated NF-κB (nuclear factor kappa-light-chain-enhancer of activated B cells) signaling, which subsequently induced the downstream expression of interleukin (IL) 1β, IL-6, and tumor necrosis factor α (TNFα) to trigger the inflammatory responses [9]. By contrast, YPFS suppressed pro-inflammatory cytokines in a lipopolysaccharide (LPS)-induced chronic inflammation model [9]. Interestingly, a duality of YPFS in modulating the expression of immunoglobulins has also been revealed in animal studies; YPFS stimulated the production of immunoglobulin after antigens were directly injected into the body, whereas YPFS suppressed immunoglobulin production when external antigens were infused in the nasal mucus [7].

Inflammatory bowel disease (IBD) is a complex group of inflammation-associated diseases involving alterations in mucosal immunity and gastrointestinal physiology. Macrophages express numerous inflammatory mediators including inducible nitric oxide synthase (iNOS) and cyclooxygenase-2 (COX-2), and both of these enzymes play pivotal roles in the pathogenesis of acute and chronic inflammation, for example in IBD [10]–[11]. The signaling messengers, NO and prostaglandins, produced by iNOS and COX-2, respectively, are required for these functions, including mucosal defense, gastric acid production, protection of epithelial cells, recruitment of leukocytes to the mucosa, release of inflammatory mediators, and vasodilation of gastric mucosa [12]–[17]. Several lines of evidence indicate that a reduction in the levels of NO and prostaglandins, which results from diminished expression of iNOS and COX-2, might lead to the damage of gastrointestinal system [18], [19]. Conversely, intestinal alkaline phosphatase (IALP), a small intestinal brush-border enzyme that provides resistance to bacterial invasion, functions as a gut mucosal defense factor [20]. Indeed, a reduced expression of IALP in IBD patients was shown to be closely correlated with gut inflammation [21], [22].

In TCM clinics today, YPFS is widely used in treating IBD; however, the underlying mechanism of YPFS remains poorly understood. In this study, we aimed to reveal the possible *in vitro* mechanism by which YPFS exerts its effect in treating IBD, and we chose 2 specific cellular models: macrophages and enterocytes. Our investigations included studying the role of YPFS in (i) modulating the expression of iNOS and COX-2 in activated and non-activated murine macrophages; and (ii) enhancing the activity of IALP in Caco-2 cells.

## Results

### Preparation of Standardized YPFS

The herbal extracts of YPFS, AR, AMR, and SR, were prepared according to the method described in our previous study [9]. Two approaches were chosen to control the quality of YPFS: chemical fingerprinting and minimal-marker requirement. The known chemicals were identified in fingerprints, and we selected 15 chemical markers from YPFS decoction: (1) AR-derived flavonoids: calycosin-7-O-β-D-glucoside, calycosin, ononin, and formononetin; (2) AR-derived saponins: astragaloside IV, III, and II; (3) AMR-derived sesquiterpenoids: atractylenolide I, II, and III; (4) SR-derived chromones prim-O-glucosylcimifugin and 5-O-methylvisammioside; and (5) SR-derived coumarins: scopoletin, isopsoralen, and psoralen (see Table 1 of Du et al., 2013). In this study, we evaluated the biological functions of these selected herbal extracts. The established chemical parameters served as the control for repeatability of the below biochemical analyses. The cytotoxicity of all the herbal extracts in cultures were tested, the results of which were consistence with our previous study [9]. Thus, the maximum dose of all extracts could be at 3 mg/mL in the following experiments.

### YPFS inhibits LPS-induced enzyme expression

**Figure 1:**
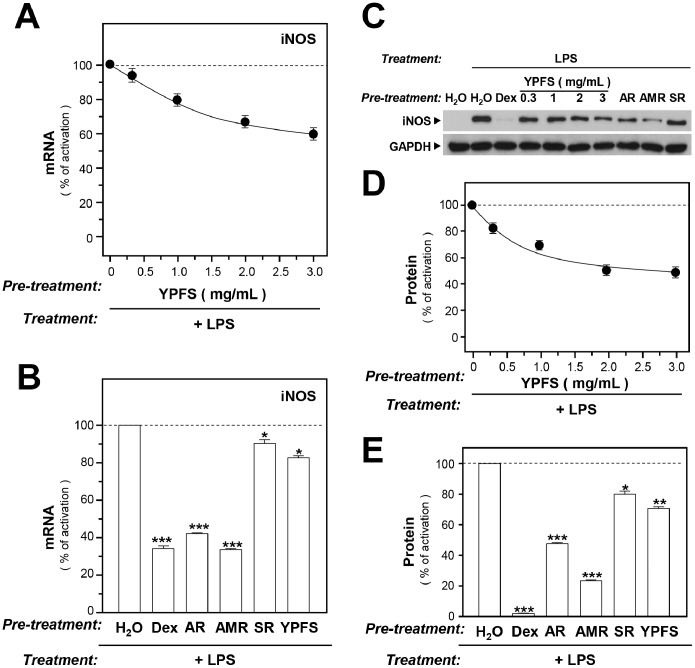
YPFS suppresses LPS-induced expression of iNOS in cultured macrophages. Cultured macrophages were treated with herbal extracts for 3 hours, after which LPS (1 µg/mL) was applied to the cultures for 24 hours to mimic chronic inflammation. Here, 10 µM dexamethasone (Dex) served as a positive control. The levels of mRNAs encoding iNOS were determined using real-time PCR (RT-PCR), performed with GAPDH serving as an internal control for normalization. The expression of iNOS protein was examined using western-blotting analysis. Whole cell lysates of macrophages were collected and equal amounts of total protein were loaded on gels and stained with an anti-iNOS antibody. (**A**): Inhibition of iNOS mRNA expression by YPFS in LPS-stimulated RAW264.7 cells. (**B**): Inhibition of iNOS mRNA expression by single herbs in LPS-stimulated RAW264.7 cells; 1 mg/mL of each of the herbal extracts was added to macrophages and the expression level of iNOS mRNA was determined using RT-PCR. (**C**): Western blots showing iNOS staining. (**D**): Inhibition of iNOS protein expression by YPFS in LPS-stimulated RAW264.7 cells. (**E**): Inhibition of iNOS protein expression by individual herbs in LPS-stimulated RAW264.7 cells. Single herbal extracts were added to LPS-stimulated macrophages for 24 hours. Results in (D) and (E) were calculated using the western blots shown in (C). Values are expressed as % LPS-induced activation; mean ± SD are shown, *n = *4, each with triplicate samples. *p<0.05; **p<0.01; ***p<0.001.

Abnormal up regulation of iNOS and COX-2 is commonly involved in several inflammation-induced diseases, e.g. IBD. We determined the inhibitory effects of YPFS on expression of iNOS in LPS-stimulated RAW 264.7 macrophages. As expected, LPS activated expression of iNOS (**Fig. 1**). However, when the cells were treated with YPFS for 3 hours before applying LPS, the expression of iNOS mRNA was inhibited in a dose-dependent manner, having a maximal effect of ∼40% inhibition (**Fig. 1A**). Next, the inhibitory effects of individual herbs were determined. The extracts of AR and AMR suppressed iNOS expression by nearly 60%, whereas SR extract exhibited only a mild suppressive effect (∼10%) (**Fig. 1B**). The positive control used here was dexamethasone **(** **Fig. 1B** **)**. In the background, the iNOS protein was barely detectable in macrophages (**Fig. 1C and 1D**), and the treatment with LPS potently induced iNOS expression (**Fig. 1C**). However, the addition of YPFS, as well as of single herbal extracts, down regulated LPS-induced iNOS protein level similarly, as in the case of mRNA expression, although a slightly larger effect was observed (**Fig. 1C and 1E**). Moreover, the suppression of iNOS expression by YPFS was in a dose-dependent manner (**Fig. 1D**).

**Figure 2:**
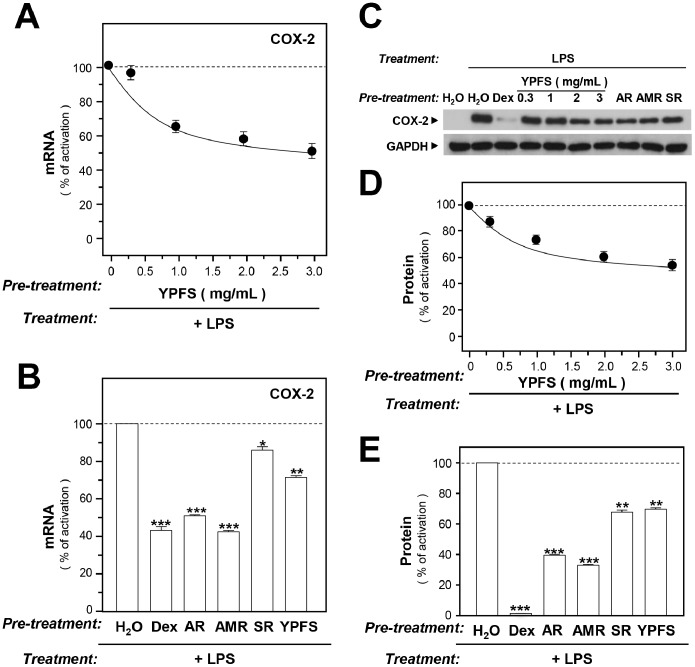
YPFS suppresses LPS-induced expression of COX-2 in cultured macrophages. Cultured macrophages were treated with herbal extracts and stimulated with LPS as described in Fig. 1 legend; 10 µM Dex was used as a positive control. The levels of COX-2 mRNA were determined using RT-PCR and normalized relative to the GAPDH signal. Lysed cells were prepared and equal amounts of proteins were subjected to western blotting performed using an antibody specific for COX-2. GAPDH was used as the internal control. (**A**): Suppression of COX-2 mRNA expression by YPFS in LPS-stimulated RAW264.7 cells. (**B**): Inhibition of COX-2 mRNA expression by herbal extracts of AR, AMR, and SR in LPS-stimulated RAW264.7 cells. (**C**): Representative western blots showing COX-2 staining. (**D**): Suppression of COX-2 protein expression by YPFS in LPS-stimulated RAW264.7 cells. (**E**): Inhibition of COX-2 protein expression by herbal extracts of AR, AMR, and SR in LPS-stimulated RAW264.7 cells. Results in (D) and (E) were calculated using the western blots shown in (C). Values are expressed as % LPS-induced activation; mean ± SD are shown, *n* = 4, each with triplicate samples. *p<0.05; **p<0.01; ***p<0.001.

COX-2, a key enzyme in the production of prostaglandins from arachidonic acid, is considered as a pro-inflammatory enzyme and a target in the treatment of inflammation [23]. Here, we determined the effect of YPFS on COX-2 expression in LPS-stimulated macrophages. Using the aforementioned treatment protocol, we co-applied the herbal extracts and LPS to cultured macrophages as to examine the expression of COX-2 mRNA and protein. LPS activated COX-2 expression (**Fig. 2**), and adding YPFS to LPS-treated macrophages potently lowered the mRNA and protein levels of COX-2; the effect occurred in a dose-dependent manner, with a maximal suppression of ∼40% (**Fig. 2A, 2C, and 2D**). As in the case of iNOS, the COX-2 protein was undetectable under the untreated conditions (**Fig. 2C**), but which was stimulated by LPS. Treatment with AR and AMR extracts suppressed, relative to control, LPS-induced expression of COX-2 mRNA by nearly 55% (**Fig. 2B**) and COX-2 protein by ∼60% (**Fig. 2E**). Moreover, treating LPS-stimulated macrophages with SR led to ∼18% and ∼30% reductions in expression of COX-2 mRNA and protein, respectively (**Fig. 2B, 2C, and 2E**). Dexamethasone again served as a positive control.

**Figure 3:**
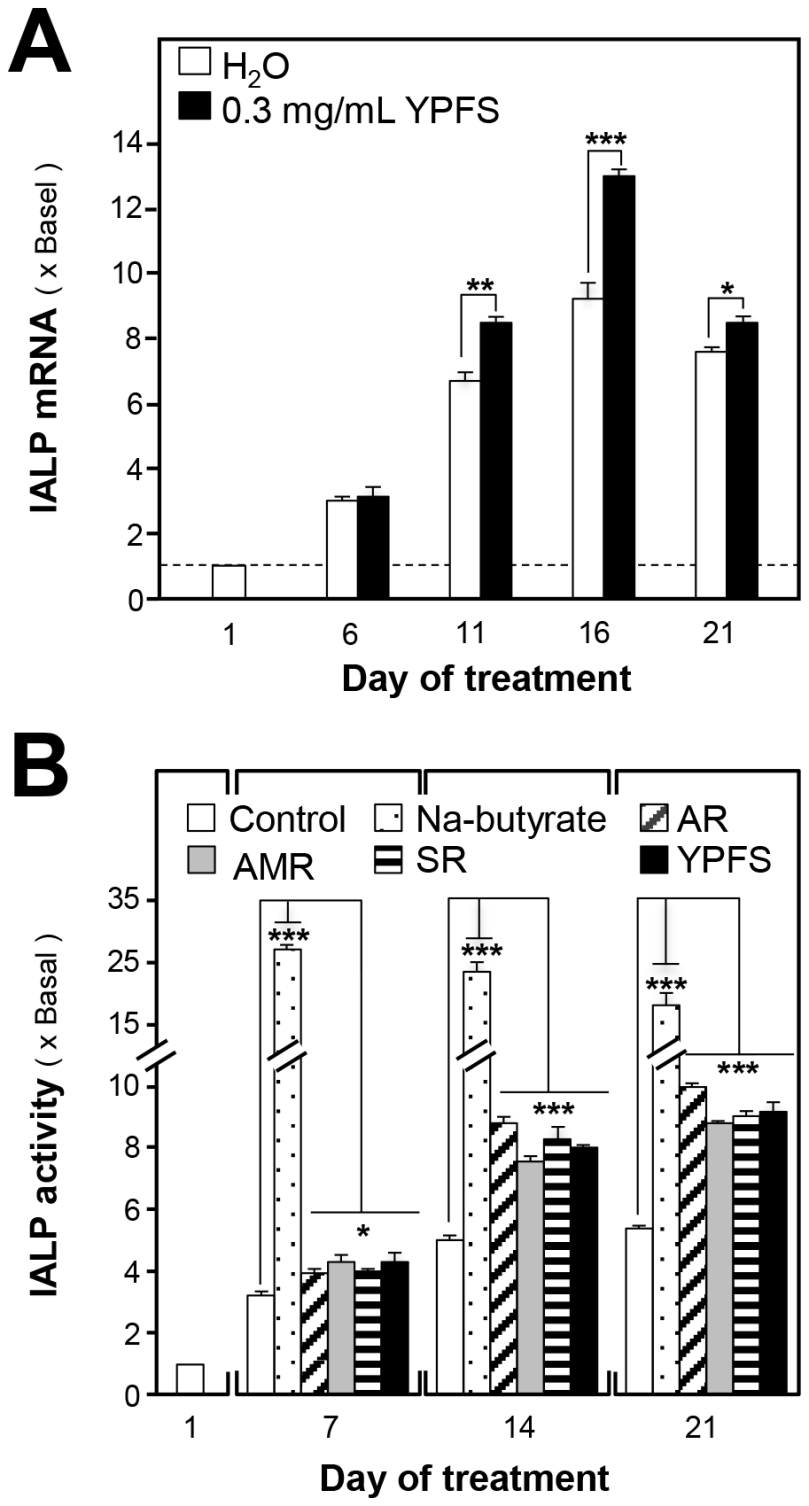
YPFS stimulates IALP expression and activity in Caco-2 cells. (**A**): Caco-2 cells (100,000/well) cultured in 6-well plates were treated with herbal extracts and cells were harvested on various days. IALP mRNA levels were quantified using RT-PCR, and the values were normalized relative to GAPDH expression. (**B**): Caco-2 cells (50,000/well) cultured in 12-well plates were treated with herbal extracts. Cells were harvested on various days as in (A) and extracts were prepared using a lysis buffer (pH 10.4). IALP activity in Caco-2 cells was measured by mixing samples with 5 mM p-nitrophenyl phosphate, and absorbance was measured at 405 nm; enzyme activity is expressed as µmol cleaved substrate/mg protein. Here, sodium butyrate (1 mM) served as the positive control. Values are expressed as fold-increases relative to basal reading (taken on the first day of culture); mean ± SD are shown, *n = *3, each with triplicate samples. *p<0.05; **p<0.01; ***p<0.001.

Caco-2 cell line acquires the structural and biochemical properties of small intestinal enterocytes after differentiation: this is an excellent model for studying intestinal epithelial cell proliferation and differentiation. We examined the expression of IALP, an indicator of colonocyte differentiation, in cultured Caco-2 cells. In water-treated cells, the expression of IALP mRNA increased within 16-day of culture; a slightly decrease could be observed in 21-day of culture (**Fig. 3A**). When Caco-2 cells were exposed to YPFS for various time intervals, the expression of IALP mRNA expression was induced, with maximal induction reaching ∼30% as compared with the negative controls in 16-day of culture (**Fig. 3A**).

We measured the enzymatic activity of IALP, and the results agreed with the mRNA expression results. The IALP activity in Caco-2 cells showed a 5–6-fold increase after 21 days of culture (**Fig. 3B**), and application of either YPFS or the single herbs in the cultures resulted in an 8-fold induction of IALP activity (**Fig. 3B**). All of the herbal treatments activated IALP to similar levels. Sodium butyrate inhibited cell proliferation and induced differentiation in mammalian cells, and which was used as a positive control: this induced a 25-fold increase in IALP activity after 7 days of treatment (**Fig. 3B**).

### YPFS induces pro-inflammatory enzymes

**Figure 4:**
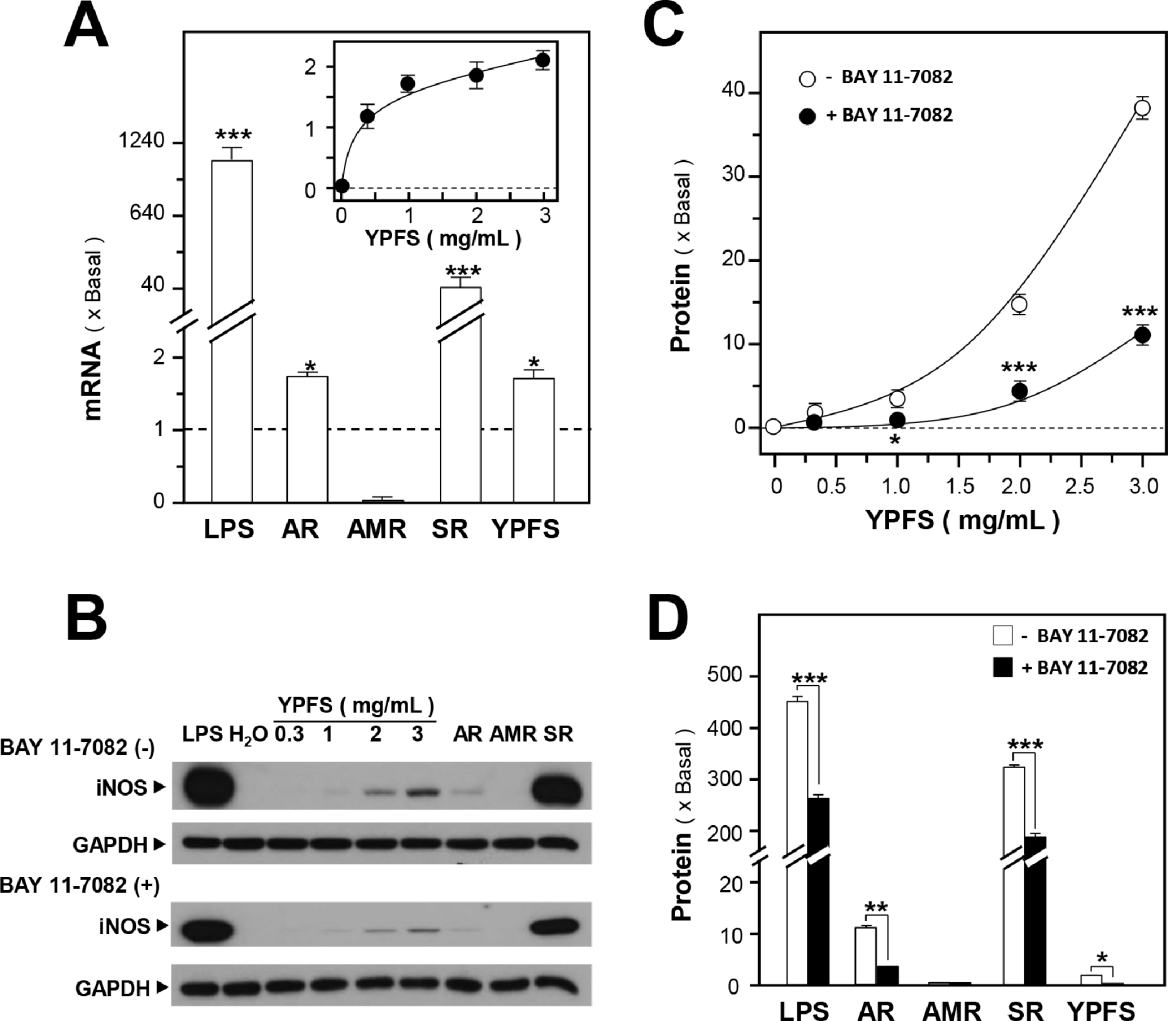
YPFS induces iNOS expression in macrophages. (**A**): Cultured macrophages were treated with 1 mg/mL of the single herbal extracts and YPFS (inset) for 24 hours, and then the expression of iNOS was determined using RT-PCR, with GAPDH serving as the internal control. LPS (1 µg/mL) was used the positive control. (**B**): Effects of herbal extracts on the expression of iNOS protein in cultured macrophages in the presence and absence of the NF-κB-specific inhibitor BAY 11-7082 (5 µM). (**C**): Macrophages were treated with YPFS for 24 hours after first incubating the cells for 3 hours with or without the NF-κB inhibitor BAY 11-7082 (5 µM). Cell lysates were collected, diluted to equal protein concentrations, and analyzed by means of western blotting. (**D**): Macrophages were treated with 1 mg/mL of the individual herbs (AR, AMR, and SR) for 24 hours after first incubating the cells with or without BAY 11-7082 (5 µM) for 3 hours. Results in (C) and (D) were calculated using the western blots shown in (B). Values were normalized using the internal control GAPDH and are expressed as fold-increases relative to basal reading (untreated cultures); mean ± SD are shown, *n* = 5, each with triplicate samples. *p<0.05; **p<0.01; ***p<0.001.

Previously, we demonstrated that YPFS exhibited a dual effect in regulating cytokine expression and, in parallel, potently activated NF-κB [9]. Here, we further investigated the inductive effects of YPFS on expression of iNOS, a downstream regulator of NF-κB. Treatment with YPFS induced iNOS expression in a dose-dependent manner, the effects of which were ∼2- and ∼40-fold of increase measured for mRNA and protein levels, respectively (**Fig. 4A, 4B, and 4C**). SR robustly induced iNOS mRNA and protein expression, almost 40 and 350 folds, respectively (**Fig. 4A, 4B, and 4D**); however, AMR did not exhibit a strong inductive effect after treatment for 24 hours, whereas AR increased iNOS mRNA expression ∼2 folds and protein expression ∼10 folds (**Fig. 4A, 4B, and 4D**). LPS induced iNOS expression markedly (**Fig. 4A, 4B, and 4D**) and served as a positive control. To further investigate the underlying mechanism of action of these herbs, we used a specific NF-κB inhibitor, Bay 11-7082. Treatment with Bay 11-7082 partially blocked YPFS-induced iNOS expression (**Fig. 4B and 4C**), and Bay 11-7082 also suppressed AR- and SR-induced iNOS expression ∼3 and ∼120 folds, respectively (**Fig. 4B and 4D**).

**Figure 5:**
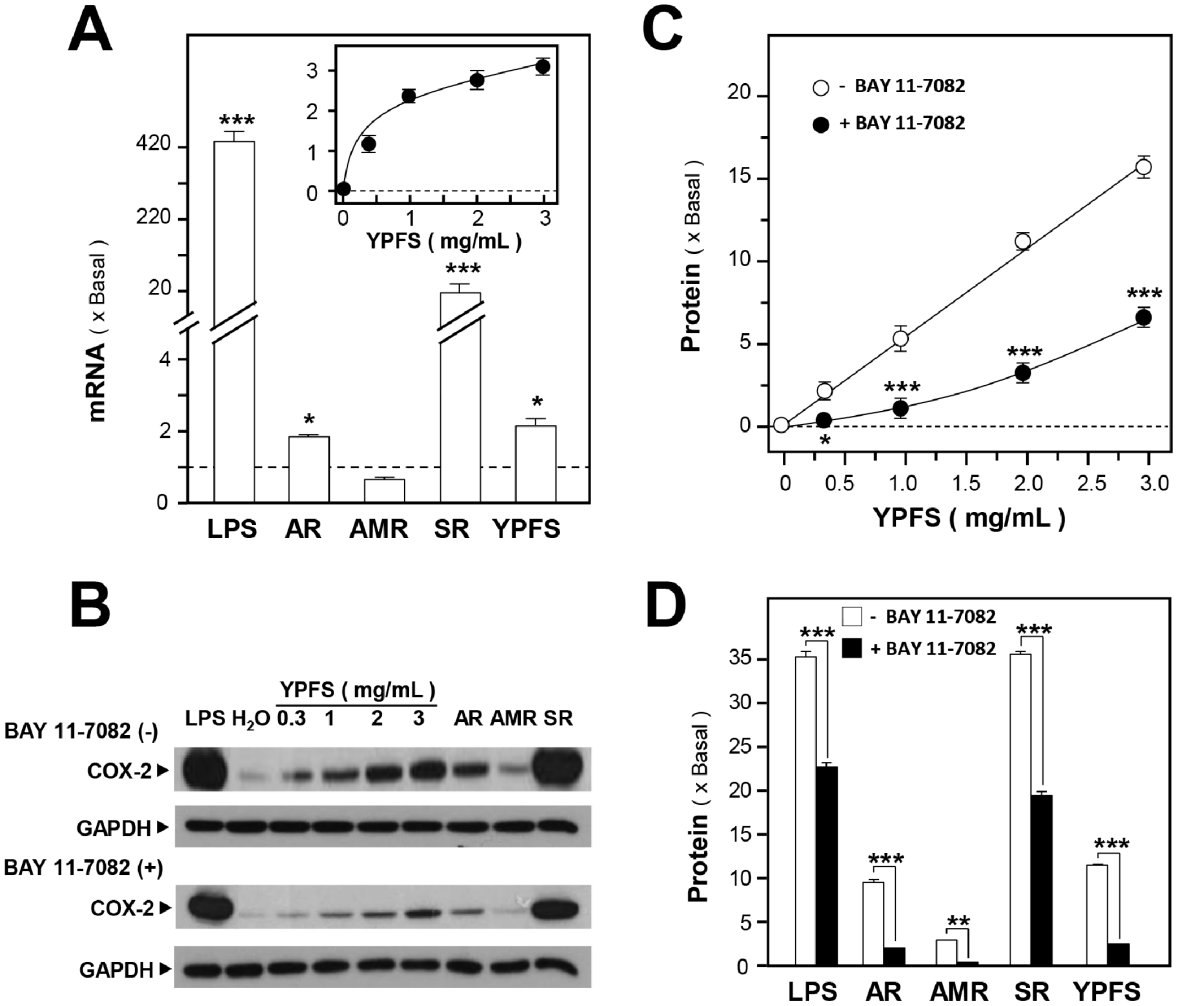
YPFS induces COX-2 expression in macrophages. (**A**): Cultured macrophages were treated with herbal extracts as described in Fig. 4 legend for 24 hours and then COX-2 mRNA expression levels were determined using RT-PCR. GAPDH was used as an internal control for normalization and LPS (1 µg/mL) was used as the positive control. (**B**): Macrophages were treated with herbal extracts for 24 hours after first incubating the cells with or without the NF-κB-specific inhibitor BAY 11-7082 (5 µM) for 3 hours. Cell lysates were collected, diluted to equal protein concentrations, and analyzed by means of western blotting. Representative western blots are shown. (**C**): Effects of BAY 11-7082 on the expression of COX-2 protein induced by YPFS in cultured macrophages. (**D**): Effects of BAY 11-7082 on COX-2 protein expression induced by individual herbs in cultured macrophages. Results in (C) and (D) were calculated using the western blots shown in (B). Values were normalized using the internal control GAPDH and are expressed as fold-increases relative to basal reading (untreated cultures); mean ± SD are shown, *n* = 5, each with triplicate samples. *p<0.05; **p<0.01; ***p<0.001.

Next, we studied the activation of COX-2 expression by YPFS. In cultured macrophages, LPS robustly induced the expression of COX-2 mRNA and protein, nearly 420 and 35 folds, respectively (**Fig. 5A, 5B, and 5D**). In these cells, YPFS treatment increased COX-2 mRNA and protein expression in a dose-dependent manner, with maximal levels reaching ∼3 and ∼17 folds, respectively (**Fig. 5A, 5B, and 5C**). In the case of treatment with single herbal extract, AR and SR increased COX-2 mRNA expression nearly 2 and 20 folds, respectively, whereas AMR did not induce COX-2 mRNA expression (**Fig. 5A**). When added separately, AR, AMR and SR increased COX-2 protein expression about 10, 3, and 35 folds, respectively (**Fig. 5B and 5D**). Similar to iNOS expression, the treatment with BAY 11-7082 partially blocked the induction of COX-2 expression (**Fig. 5B, 5C, and 5D**). Thus, the herbal extracts used in this study might stimulate COX-2 expression through the NF-κB signaling pathway.

## Discussion

The complexity of TCM herbal decoctions is a major obstacle in the internationalization of Chinese medicine, and the complexity hinders the discovery of the mechanisms by which TCM functions as a therapeutic agent in disease treatment. In our laboratory, a systematic approach to study TCM formulae has been successfully developed using distinct methods based on chemical and biological assessments. In the last few years, we have used these methods to standardize herbs, to verify herbal formulae, and to study the mechanisms of action of various herbal decoctions, e.g. Danggui Buxue Tang, Fo Shou San, and Kaixin San [24]–[27]. These systematic approaches were applied in this study on YPFS to uncover the underlying mechanism in cultures by which YPFS functions in clinical treatment of IBD.

The gastrointestinal tract is the largest and most complex immune environment in the body. IBD is a chronic, relapsing, inflammatory disorder of the gastrointestinal tract, the pathogenesis of which is manifested as complex and multifactorial processes, and which is classified as autoimmune disorder. Non-steroidal anti-inflammatory drugs (NSAIDs) are effective anti-inflammatory agents that are widely used to treat inflammation-associated diseases. However, using NSAIDs to inhibit the expression of iNOS and COX-2 can lead to severe gastrointestinal damage [19]. Thus, NSAIDs is avoided for the treatment of IBD. Searching for effective drugs is urgent needed for the drug development of therapy and prevention of IBD. Excessive iNOS and COX-2 expression is associated with various forms of gastrointestinal mucosal inflammation, whereas low levels of iNOS and COX-2 have been reported to play protective roles in IBD [16], [28]–[30]. Thus, to investigate the mechanism by which YPFS functions, we evaluated the duality of YPFS in modulating the expression of iNOS and COX-2. In activated or non-activated macrophages, YPFS could play a modulatory role in balancing the expression of iNOS and COX-2. The regulation of these enzymes might offer a means by which YPFS could be used as a suitable therapeutic approach to treat IBD, in particularly, when the intake of anti-inflammatory agents NSAIDs cannot be avoided.

YPFS contains AR, AMR, and SR, and the activities of these 3 herbs have been reported. In macrophages, AR extracts stimulated the expression of pro-inflammatory cytokines (IL-1β, IL-6, and TNFα) [9], whereas AR extracts or AR fraction can also suppress the expression of these pro-inflammatory cytokines [9], [31]. Moreover, an AMR-derived glycoprotein was shown to markedly stimulate the production of TNFα in splenocytes [9], [32], whereas AMR-derived atractylenolide I and III were shown to exert anti-inflammation effects by suppressing LPS-induced expression of TNFα in cultured macrophages [9], [33]. SR extracts, as shown in this study, strongly upregulated iNOS expression, but three SR-derived active ingredients, anomalin, imperatorin and deltoin, can inhibit the induction of iNOS in LPS-activated macrophages [34]–[35]. In the complex immune system, a balance must be struck between the activation and suppression of mediators such as pro-inflammatory cytokines, iNOS, and COX-2. Thus, the duality of YPFS in modulating these mediators could represent a unique property that helps maintain the hemostasis of the immune system.

To understand the mechanism underlying the duality of YPFS in regulating immune responses, we investigated the effects of YPFS in the IL-10 anti-inflammatory signaling pathway. Although YPFS activated the expression of the anti-inflammatory cytokine IL-10 in macrophages after treatment for 24 hours, YPFS suppressed IL-10 expression in LPS-stimulated macrophages in a dose-dependent manner, indicating that the anti-inflammatory effects of YPFS might not be mediated through the IL-10 pathway (Du et al., unpublished data). The mechanism responsible for the dual role played by YPFS-induced signaling in the immune system thus remains unknown, but studies on ursolic acid could offer valuable clues. Ursolic acid, a natural pentacyclic triterpenoid carboxylic acid, exhibits contrasting anti-inflammatory and pro-inflammatory bioactivities *in vitro* and *in vivo* [36]–[40] effects that are similar to those of YPFS. However, only limited *in vitro* and *in vivo* studies have been conducted on the mechanisms of ursolic acid action. Thus, further investigation into the mechanistic underpinnings of the duality YPFS action in modulating immune functions should provide new insights into this property. More importantly, the mechanism responsible for this dual effect of YPFS must be studied comprehensively to determine the risks and benefits of clinically administering this herbal decoction.

## Materials and Methods

### Chemicals and reagents

LPS, dexamethasone, Bay 11-7082, and p-nitrophenyl phosphate (pNPP) were purchased from Sigma (St. Louis, MO); all chemicals were >98% pure. Various culture media and supplements were obtained from Invitrogen Technologies (Carlsbad, CA). Fetal calf serum was from Hyclone (Thermo Fisher Scientific, Waltham, MA). Antibodies against iNOS and COX-2 were purchased from Abcam (Cambridge, UK) and Cayman (Ann Arbor, MI) respectively. The antibody against glyceraldehyde 3-phosphate dehydrogenase (GAPDH) was obtained from Abcam Ltd. The enhanced chemiluminescence (ECL) reagent was purchased from Amersham Biosciences (Piscataway, NJ). Penicillin, streptomycin, and horseradish peroxidase (HRP)-conjugated anti-mouse secondary antibodies were purchased from Invitrogen Technologies.

### Plant materials and preparation of herbal decoctions

The roots of *A. membranaceus* var. *mongholicus* (AR), the rhizomes of *A. macrocephala* (AMR), and the roots of *S. divaricata* (SR) were collected from Shanxi, Anhui, and Heilongjiang provinces of China, respectively. The plant materials were authenticated by one of the authors, Dr. Tina Dong, and the corresponding voucher specimens, in the form of whole plants, were deposited at Center for Chinese Medicine at The Hong Kong University of Science and Technology. The raw materials were purchased from medicinal herbal markets. No special permissions were required for visiting the collection sites or for conducting activities related to collecting the raw materials, and the locations were not privately owned or protected. The herbal extracts were prepared using methods described in our previous study [9]. Typically, the crude materials (in slices) of AR, AMR, and SR were weighed according to the weight ratio of 1∶2∶1. The herbal mixture was boiled in 8 volumes of water (v/w) with moderate heating for 2 hours, and the residues were re-boiled in 6 volumes of water for 1 hour. The 2 extracts were pooled and filtered, dried through by lyophilization, and stored at 4°C for use in studies.

### Culture of RAW 264.7 murine macrophages

RAW 264.7 murine macrophages, obtained from American Type Culture Collection (ATCC; Manassas, VA), were cultured in high-glucose Dulbecco’s Modified Eagle’s medium (DMEM) supplemented with 10% heat-inactivated FBS, 100 U/mL penicillin, and 100 µg/mL streptomycin and maintained in a humidified CO_2_ (5%) incubator. When the cells reached 80% confluence, they were harvested by scraping into 10 mL of medium. To investigate the effects of the herbal extracts on the expression of iNOS and COX-2 genes, we used Raw 264.7 cells cultured in 6-well plates at a density of 250,000 cells/well. To test the effects of the extracts on iNOS and COX-2 protein expression, 80,000 Raw 264.7 cells/well were cultured in 12-well plates and treated with reagents for 24 hours; cells lysates were then prepared and subjected to western-blotting analyses.

### Culture of Caco-2 cells

Caco-2 cells (from ATCC) were grown in Eagle’s MEM (EMEM) supplemented with 20% FBS, 1 mM sodium pyruvate, 100 U/mL penicillin, and 100 µg/mL streptomycin and cultures were maintained in a 5%-CO_2_ incubator at 37°C. After the cells reached 80% confluence, they were harvested using a 0.25% trypsin–EDTA solution. We cultured 100,000 Caco-2 cells/plate in 6-well plates, treated the cells with reagents on all days except the first day of culture, and harvested the cells on days 1, 6, 11, 16, and 21. In these experiments, we investigated the effects of herbal extracts on the expression of the IALP gene. In another set of assays to analyze IALP activity, 50,000 cells/well were cultured in 12-well plates and then treated with herbal extracts every day starting 24 hours after seeding the cells. Cell lysates were collected on days 1, 7, 14, and 21, and IALP activity was measured.

### Quantitative real-time PCR

To analyze the mRNA expression of iNOS and COX-2 in cultured macrophages and of IALP in Caco-2 cells, cultures were treated with herbal extracts. Total RNA was isolated using TRIzol reagent and reverse transcribed into cDNAs according to the manufacturer’s instructions (Invitrogen). Real-time PCR was performed using the SYBR Green Master mix and ROX reference dye according to the manufacturer’s instructions (Applied Bioscience, Foster city, CA). The primers were the following: 5′- GCC CTG CTT TGT GCG AAG TGT CAG -3′ and 5′- GCA CCT GGA ACA GCA CTC TCT TG -3′ for murine iNOS (254 bp; NM_010927.3); 5′- GGT TGC TGG GGG AAG AAA TGT GCC -3′ and 5′- GAC GAG GTT TTT CCA CCA GCA GGG -3′ for murine COX-2 (242 bp; NM_011198.3); and 5′- GGT ATG TGT GGA ACC GCA CTG AG -3′ and 5′- GAA CAT GAC CGC CTC AGT GAG TG -3′ for human IALP (269 bp; NM_001631.3). GAPDH was used as an internal control in all cases, and its primer sequences were 5′- AAC GGA TTT GGC CGT ATT GG-3′ and 5′- CTT CCC GTT CAG CTC TGG G-3′ (657 bp; NR_0215885). The SYBR Green signal was detected using the Mx3000ptm multiplex quantitative PCR machine from Stratagene (La Jolla, CA). Transcript levels were quantified and the values measured for the target genes were normalized relative to that of GAPDH expression in the same sample before being compared. To confirm the specific amplification of PCR products, gel electrophoresis and melting curve analysis were performed.

### Western-blotting analysis

Macrophages were treated with reagents for 24 hours and then the cultured cells were collected in a low-salt lysis buffer (50 mM HEPES (pH 7.5), 250 mM NaCl, 10% glycerol, 1% Triton, 1.5 mM MgCl_2_, 1 mM PMSF, 1 mM EGTA, 2 mM Na_3_VO_4_, and 10 mg/mL each of aprotinin and leupeptin). The collected cell lysates were vortexed for 10 min and the insoluble cell debris were removed by centrifugation. Total protein concentrations were measured using the Bradford method and then all lysates were diluted to the same concentration. The cell lysates were boiled in a gel-loading buffer (20% glycerol, 10% β-mercaptoethanol, 6% SDS, 125 mM Tris–HCl, pH 6.8, 0.005% Bromophenol Blue; 1∶5) at 95°C for 10 min. The proteins extracted from treated and untreated cells were separated using SDS-PAGE (8% gels) and then electro-blotted onto nitrocellulose membranes. The membranes were incubated in a blocking solution (5% skim milk) for 2 hours at room temperature, and then incubated overnight at 4°C with primary antibodies (1∶1,000 dilution to detect the induction of iNOS and COX-2, 1∶10,000 to detect the suppression of iNOS and COX-2, and 1∶50,000,00 to stain for GAPDH). After washing 4 times with Tween-20/Tris-buffered saline (TBS-T), the membranes were incubated with secondary antibodies for 1 hour at room temperature (2 hours in the case of iNOS induction); the dilutions used were 1∶10,000 HRP-conjugated anti-mouse antibody to detect GAPDH; 1∶2,500 and 1∶10,000 HRP-conjugated anti-mouse antibody to detect iNOS induction and suppression, respectively; and 1∶2,500 and 1∶10,000 HRP-conjugated anti-rabbit antibody to detect COX-2 induction and suppression, respectively. Blots were washed 4 times with TBS-T and then developed using the ECL method. The intensities of immunoreactive bands in the control and various treated samples, run on the same gel under strictly standardized ECL conditions, were compared using an image analyzer, with a calibration plot being constructed using a parallel gel in which serial dilutions of one of the samples was run.

### Intestinal alkaline phosphatase assay

Caco-2 cells (50,000/well) cultured in 12-well plates were treated with reagents every day starting 24 hours after plating, and cells were harvested on days 1, 6, 11, 16, and 21. Cultures were collected using a lysis buffer containing 100 mM potassium phosphate buffer, 0.2% Triton X-100, and 1 mM dithiothreitol (pH 10.4). IALP activity in Caco-2 cells was measured by mixing the samples with 5 mM pNPP in an IALP buffer (pH 10.4) containing 0.1 M glycine, 1 mM MgCl_2_, and 1 mM ZnCl_2_. After incubating the samples at 37°C for specified times, absorbance was measured at 405 nm to determine enzyme activity, which was expressed as µmol cleaved substrate/mg protein.

### Statistical analysis and other assays

Statistical analyses were performed using one-way ANOVA followed by the Student’s *t* test. Statistically significant changes were classed as [*] for *p*<0.05, [**] for *p*<0.01, and [***] for *p*<0.001. Protein concentrations were determined in 96-well microtiter plates by following the instructions of Bradford’s method and using a kit from Bio-Rad Laboratories (Hercules, CA). Typically, one part of concentrated dye reagent was diluted with 4 parts of double-distilled water and mixed thoroughly before use; 6 dilutions of BSA standard (0.05–0.5 µg/mL) were used in the tests and samples were assayed in triplicate. The concentrations of proteins were determined from the standard curve.
